# Impact of nasal photodisinfection on SARS-CoV-2 infection in an industrial workplace

**DOI:** 10.1016/j.puhip.2023.100393

**Published:** 2023-05-30

**Authors:** Richard Rusk, Judy Hodge

**Affiliations:** a Department of Emergency Medicine, Rady Faculty of Health Sciences, University of Manitoba, Winnipeg, Manitoba, Canada; b Katrime Integrated Health, Winnipeg, Manitoba, Canada

**Keywords:** COVID-19, Occupational safety, Antimicrobial photodisinfection therapy, Essential workers, Nasal decolonization, Infection control, Broad spectrum antimicrobial

## Abstract

**Objectives:**

We aimed to evaluate a quality improvement initiative designed to control SARS-CoV-2 (COVID) using the large-scale deployment of antimicrobial photodisinfection therapy (aPDT) for nasal decolonization in a Canadian industrial workplace (a food processing plant).

**Study design:**

Using a retrospective chart review of treatment questionnaires, linked to COVID laboratory testing results, a quality improvement assessment was analyzed to determine treatment effectiveness and safety.

**Methods:**

This voluntary aPDT intervention involved the administration of a light-sensitive liquid to the nose followed by nonthermal red-light irradiation on a weekly basis. Employees in food processing industries are at increased risk for COVID infection due to the nature of their work environments. In an effort to mitigate the transmission and consequences of the disease among such workers and the community at large, aPDT was added to a well-established bundle of pre-existing pandemic safety measures (e.g., mask-wearing, testing, contact tracing, workplace-engineered barriers, increased paid sick leave).

**Results:**

From December 2020 to May 2021, we found high interest in and compliance with aPDT treatment, along with a statistically significant lower PCR test positivity rate in the study population in comparison to the case rates for the local Canadian province. Treatment safety monitoring and outcomes of the aPDT program demonstrated no serious adverse events.

**Conclusions:**

This study suggests nasal photodisinfection provides safe and effective COVID viral suppression when deployed across the majority of workers in an industrial workplace setting.

## 1 Introduction

SARS-CoV-2 (COVID-19) outbreaks are known to cause significant adverse outcomes, including worker absenteeism, supply chain interruptions, and acute and long-term human illnesses, hospitalizations, and deaths [1]. Beginning in 2020, medical providers and employers throughout the world rapidly implemented enhanced safety protocols in response to the SARS-CoV-2 pandemic, such as personal protective equipment (PPE), social distancing, workplace-engineered barriers, and improved handwashing [2]. Despite these interventions, the impact of the first waves of the pandemic dramatically threatened human and animal health when COVID-19 disproportionately affected essential employees within the food processing (e.g., poultry, pork, beef) industry [3,4]. Staff working in food processing facilities remain at increased risk for SARS-CoV-2 transmission based on the duration and type of work interactions with other employees, such as being in close contact on processing lines, in cold temperatures, and within enclosed work environments during multi-hour shifts [5].

Since the onset of the pandemic, meat processing plants have reported a disproportionately large number of employees contracting COVID-19,^2^ requiring these plants to shut down operations. Furthermore, the presence of slaughtering plants in a community has been found to be associated with 400–600 additional COVID-19 cases per hundred thousand from the baseline rate, and an increase in the death rate by 7–10 deaths per hundred thousand, or 37 to 50% over the baseline rate [6]. However, it has been documented that transmission in the workplace decreases as safety interventions are implemented [1,3]. Based on this and similar research studies, several Canadian workplaces added nasal photodisinfection to their protection protocols as an emergency response to the pandemic and in consideration of the documented safety and efficacy of the treatment [7].

Nasal photodisinfection, known more specifically as antimicrobial photodisinfection therapy (aPDT), has been investigated as an adjunct to these interventions to further mitigate the potential impact of COVID-19 outbreaks within the workplace and surrounding communities, including the potential impact of asymptomatic transmission [8]. aPDT involves the application of a topical photosensitizer inside the nose, which has a positive charge that preferentially binds to negatively-charged microorganisms. Two small nasal cones connected to a light source are then inserted into the nares (nostrils) to activate the photosensitizer using a specific wavelength of red light. During illumination, the excited photosensitizer reacts with nearby oxygen which generates reactive oxygen species (ROS) that destroy a broad spectrum of microbes including bacteria, viruses, and fungi. This treatment is painless and takes approximately 5 min to administer.

aPDT has been used in Canadian hospitals since 2011 [7] and has proven effective in the destruction of SARS-CoV-2 at the genomic (RNA) level [[9], [10], [11], [12]], as well as against spike proteins and receptor binding domains, both without adversely affecting human cells. A recent clinical trial using aPDT therapy on SARS-CoV-2 positive patients yielded a 90% reduction in infectivity of these cases [13]. In hospital presurgical deployment studies, aPDT intranasal therapy has been shown to result in dramatically-improved surgical outcomes (e.g., reduction of surgical site infections) [7]. Unlike traditional antimicrobials, aPDT does not lead to the development of resistance in targeted pathogens.

In this study, we evaluated a novel, large-scale deployment of nasal photodisinfection (Steriwave™ ND, Ondine Biomedical Inc., Vancouver, Canada) beginning in July 2020. Nasal photodisinfection technology was administered to employees in Canadian workplace settings in addition to other SARS-CoV-2 safety measures recommended by the U.S. Centers for Disease Control and Prevention (CDC) that had been implemented since the start of the pandemic [5]. This manuscript documents a retrospective, industrial workplace quality assurance program analysis that received Canadian ethics review board approval (HS25575 H2022:220).

## 2 Methods

Beginning July 16, 2020, aPDT was offered to a group of approximately 1500 employees operating in a single food processing plant in western Canada. Enrollment was voluntary and all participants were provided with a written consent document outlining the protocol. Signed consent forms were obtained from each individual and were stored securely within an electronic medical record (QHR Technologies Inc., Accuro® Electronic Medical Software). Processing plant management instituted stringent protocols to ensure employees were properly informed and safeguarded during the consent process, including education, question and answer sessions, pamphlet distribution, access to clinical assessments, understanding of their ability to discontinue participation at any time, and ongoing informed consent measures. Volunteers were given an incentive to encourage compliance with a bundle of infection prevention efforts, including aPDT, that consisted of fifty Canadian dollars per week. Employees were treated with aPDT weekly (on the same day and at approximately the same time each week) in consideration of clinical and operational guidance. All treatments were free of charge and completed during paid work time. Accessibility to the aPDT treatments was widespread in order to maximize ethical access; adherence levels were used to evaluate efficacy, the importance of compliance across the workforce, and the role of incentives in increasing participation. Target employee compliance with aPDT was 90% by September 15, 2020; the plant reached 75% adherence as of October 29, 2020. Prior to and running concurrently with this intervention, the plant also proactively implemented multiple safety measures that became the standard within the food processing industry [2,5]. For example, beginning in March 2020, increased paid sick leave, additional outdoor break rooms, third-party cleaning teams to disinfect high-touch surfaces three times per shift (after each break), engineered barriers, testing, social distancing during breaks, and pre-shift temperature and health screening were implemented. Furthermore, during the same period, participants were encouraged to continue to maintain high compliance with all CDC-recommended safety measures through the use of reminders, internal education videos, and trained staff available to answer any questions or concerns.

Data collected included health screenings for symptoms of active SARS-CoV-2 infection, SARS-CoV-2 test results of suspected cases, aPDT treatment frequency, adverse events/side effects from aPDT, and employee satisfaction measures. Employee satisfaction results are outside of the scope of this manuscript; however, the treatment was shown to be well received by the employee volunteers (J. Hodge, personal communication, 2021). Furthermore, participant questionnaires were completed before and one week after every aPDT session to monitor for any adverse events during treatment or within the 24-h period after treatment. Data were stored in an accredited, secure electronic medical record (QHR Technologies Inc., Accuro ® Electronic Medical Software) and within an employee database using unique identifiers to maintain anonymity and protect personal data.

## 3 Statistical analysis

SARS-CoV-2 polymerase chain reaction (PCR) test positivity data from individuals experiencing symptoms (e.g., fever, cough) who worked at the food processing plant were considered for this analysis. For privacy protection, individuals' treatment and testing data were linked using unique identifiers that replaced the need for sensitive patient information within the dataset. COVID-19 test positivity rates were calculated using the total number of positive tests (numerator) compared to the total number of positive and negative tests (denominator). Any tests with inconclusive results (n = 19) were not included in the calculation of the positivity rate. Exact binomial 95% confidence intervals (CIs) for test positivity were determined, and the statistical significance of the test positivity rates in the food plant were calculated using a Fisher's Exact Test. Also considered in this analysis were PCR test positivity rates for the same time periods from the entire Canadian province in which the plant was located. These data were downloaded from the public access database on April 1, 2022 [14]. All analyses were retrospective and were conducted in SAS version 9.4 and Microsoft Excel.

## 4 Results

Table 1 demonstrates the COVID-19 PCR test positivity rates among the food processing plant workers from December 16, 2020 to May 1, 2021 as compared to PCR test positivity rates for the entire province for the same date range.

**Table 1:** Comparing test positivity rates between workplace and overall province (Dec 16, 2020 to May 1, 2021).

Workplace (n = 558)		Province (n = 273,538)		P value
n (%)	95% CI	n (%)	95% CI	
3 (0.5)	0.1–1.6	17,473 (6.4)	6.3–6.5	<0.0001

The total number of PCR tests in the workplace totaled 558; the total number of PCR tests in the province totaled 273,538. Among the plant workers, there were three positive tests, representing 0.5% 95% CI [0.1, 1.6] of the total tests conducted in the time period. In the province as a whole, there were 17,473 positive tests, representing 6.4% 95% CI [6.3, 6.5] of the total tests conducted in the time period. A statistically significant difference (p < 0.0001) in the test positivity rate for the food processing plant workers (0.5%) versus the overall province (6.4%) was identified. This study period was chosen to capture data from points at which full aPDT compliance (∼75%) was reached within the workplace population, the second wave of the pandemic was ongoing but the third wave of the pandemic had not yet occurred, and employees had access to on-site PCR testing with results available within 24 h from a private, fully-accredited diagnostic laboratory.

Table 2, Table 3 illustrate all adverse events (side effects) in the workplace population reported by surveys conducted during treatment in real-time and one week prior (retrospectively) for the period within 24 h after aPDT. These data were collected from December 16, 2020, to May 1, 2021. Side effects were stratified by those that were expected and unexpected.

**Table 2:** Survey responses of side effects reported during and within 24 h of treatment with antimicrobial photodisinfection therapy.

	During Treatment (n = 21,459), n (%)	Within 24 Hours (n = 21,261), n (%)	Total, n (%)
Survey Responses			
Reported side effects^a^	86 (0.4)	738 (3.5)	
No side effects	21,373 (99.6)	20,523 (96.5)	
Total	21,459 (100.0)	21,261 (100.0)	
**Side Effects Reported**			
Expected	61 (0.3)	712 (3.5)	773 (3.6)
Unexpected	30 (0.1)	248 (1.2)	278 (1.3)
Total	91 (0.4)	960 (4.7)	1051 (4.9)

a Reported side effects (86, 738) are less than total side effects reported (91, 960) as some respondents indicated having multiple effects from one treatment.

**Table 3:** Survey responses of expected versus unexpected side effects reported during and within 24 h of treatment with antimicrobial photodisinfection therapy.

	During Treatment (n = 21,459), n (%)	Within 24 Hours (n = 21,261), n (%)	Total, n (%)
Expected Side Effects			
Runny nose	14 (0.1)	333 (1.6)	347 (1.6)
Sneeze	17 (.1)	234 (1.1)	251 (1.2)
Nose irritation^a^	8 (0.0)	24 (0.1)	32 (0.1)
Throat irritation	2 (0.0)	5 (0.0)	7 (0.0)
Itchy nose	15 (0.1)	53 (0.3)	68 (0.3)
Nasal congestion		39 (0.2)	39 (0.2)
Itchy throat	4 (0.0)	17 (0.1)	21 (0.1)
Odd smell/taste		6 (0.0)	6 (0.0)
Warm feeling	1 (0.0)	1 (0.0)	2 (0.0)
Total	61 (0.3)	712 (3.5)	773 (3.6)
**Unexpected Side Effects**			
Headache	1 (0.0)	101 (0.5)	102 (0.5)
Dry nose	15 (0.1)	78 (0.4)	93 (0.4)
Dry throat	4 (0.0)	29 (0.1)	33 (0.2)
Nose bleed	9 (0.0)	16 (0.1)	25 (0.1)
Dizzy		4 (0.0)	4 (0.0)
Red/blood-tinged mucus	1 (0.0)	9 (0.0)	10 (0.0)
Furunculosis		4 (0.0)	4 (0.0)
Uncomfortable		1 (0.0)	1 (0.0)
Increased acne		2 (0.0)	2 (0.0)
Anxiety		1 (0.0)	1 (0.0)
Shortness of breath		1 (0.0)	1 (0.0)
Stomachache		1 (0.0)	1 (0.0)
Triggered sinusitis		1 (0.0)	1 (0.0)
Other - unspecified	1 (0.0)	2 (0.0)	3 (0.0)
Total	30 (0.1)	248 (1.2)	278 (1.3)

a Nose irritation includes three instances in which respondent indicated “redness in the nose” within 24 h after treatment.

The majority of surveys indicated employees experienced no adverse response during treatment (99.6%) or within 24 h after treatment (96.5%). Of the total surveys (n = 21,459) collected during aPDT treatment, 86 (0.4%) indicated some reaction. Of the responses collected retrospectively after treatment (21,261), 738 (3.5%) indicated some mild reaction. The most common, expected treatment-related side effects were runny nose, sneeze, and itchy nose. The most common, unexpected side effects were headache, dry nose, and dry throat. No severe adverse reactions from aPDT were reported and no treatments were discontinued due to adverse events. Expected and unexpected treatment-related side effect characterizations are based on approximately ten years of historical Canadian use of aPDT in other healthcare settings.

## 5 Discussion

This is the first published study investigating aPDT coupled with an industry-standard bundle of interventions to evaluate COVID-19 test positivity rates of employees in a large commercial food processing operation. Among participants, treatment was well-received and voluntary compliance was high. The results in Table 1 indicate a statistically-significant decrease in test positivity rates when compared to those in the greater Canadian province. This preliminary study suggests that deploying aPDT in a commercial environment with high compliance among workers could decrease SARS-CoV-2 test positivity rates, decrease the incidence of disease among workers during times of high transmission, and potentially decrease the spread of disease in the larger community. Furthermore, aPDT has been shown to be effective against all SARS-CoV-2 variants as well as other viruses [[9], [10], [11], [12], [13],^15^]; therefore, the technique may prove to be of significant public health benefit as the COVID-19 pandemic evolves alongside future waves of viral illnesses (e.g., influenza). While certain factors of transmission may be modified to reduce the risk of COVID-19, food processing plant employees are subject to close contact for extended periods of time, creating a high-risk environment. As a result, precautions that may be effective in a community setting could be less impactful in food processing plants, which may necessitate additional safety measures [[16], [17], [18]]. Based on the method of SARS-CoV-2 transmission and the nose being a primary point of entry [15], routine nasal decolonization with aPDT may be an effective method to enhance standard SARS-CoV-2 safety measures such as high-quality masks. In this analysis, we evaluated aPDT that was deployed on an emergency basis in an attempt to administer a safe and efficacious intervention [7] to a vulnerable population (essential workers) at increased risk of death and disease who were presented with no approved alternatives before vaccinations were available.

Although no single intervention can adequately protect workers from COVID-19 [18], by offering — and incentivizing — a bundle of interventions, including novel aPDT treatment, employers may be able to better protect their employees, continue the production and distribution of food products production without disruption, and help safeguard the community at large. This kind of mitigation effort could have broader implications to other employee demographics, industries, and countries.

## 6 Limitations

This study includes certain limitations related to the retrospective design. Also, conclusions were drawn from data at a single industrial plant in Canada, and thus are not generalizable to the larger commercial footprint across Canada. The sample size was relatively small and should be increased in future studies across larger populations. While the present study demonstrated no significant relationship of covariates, such as age, gender, ethnicity, or treatment number to outcomes, potential confounding factors should be addressed in future studies. Furthermore, while industry standard SARS CoV-2 safety measures were established prior to the introduction of aPDT, the impact of the additional nasal photodisinfection can only be associated with the outcomes. Also, while other food processing plants that did not deploy aPDT continued to experience outbreaks, a direct causal relationship associated with the addition of aPDT was not definitively concluded. Lastly, employees were aware they were being observed and were incentivized to adhere to all safety measures which could have increased compliance and led to the Hawthorne effect (participant observation awareness).

## 7 Conclusion

Results of this study indicate intranasal aPDT added to a bundle of workplace safety interventions potentially suppressed the incidence of COVID-19 cases in an industrial food processing setting during the height of the SARS-CoV-2 (Delta variant) pandemic. No significant adverse events were detected among 21,261 questionnaires administered after treatment, and the process was well-tolerated with high (75%) terminal compliance. Additionally, this intervention may have had broader implications for the surrounding community by mitigating SARS-CoV-2 test positivity among plant workers when protocol compliance was maintained. It is widely documented that food processing industry essential workers are disproportionately affected by COVID-19 infection [6,16,17]. Thus, an important implication from the findings in this study is that enterprise-to-community transmission may have been reduced, preventing acute and long-term illness, disability, and death in plant employees, their families, and the broader community. This type of mitigation effort could have broader implications to other employee demographics, industries, and countries and further studies are warranted.

## Author contributions

This was a collaborative effort between the two authors, with contributions for all aspects being shared: Conceptualization, Rusk. and Hodge.; methodology, Rusk. and Hodge.; software, Rusk.; validation, Rusk. and Hodge.; formal analysis, Rusk.; resources, Rusk. and Hodge.; data curation, Rusk and Hodge.; writing—original draft preparation, Rusk. and Hodge.; writing—review and editing, Rusk. and Hodge.; visualization, Rusk. and Hodge.; supervision, Rusk.; project administration, Hodge. All authors have read and agreed to the published version of the manuscript.

## Funding

Funding was internal from personal financing by the authors, through the contribution of their time and collaboration. Hodge is a salaried employee of the food processing company.

## Statement of ethical approval

Institutional Review Board approval for this study was received from the University of Manitoba.

## Informed consent statement

Written informed consent was obtained from employees receiving Steriwave™ aPDT treatment and from employees who were tested by PCR for SARS-CoV-2 through company-provided on-site COVID-19 testing.

## Declaration of competing interest

The authors declare that they have no known competing financial interests or personal relationships that could have appeared to influence the work reported in this paper.
